# Food insecurity in adults living with severe mental illness in Australia: an exploration of causes and experiences using a co-design approach

**DOI:** 10.1007/s00127-026-03082-8

**Published:** 2026-04-29

**Authors:** Oliver Ardill-Young, Catherine O’Donnell, Alyssa Milton, Philip B. Ward, Jackie Curtis, Scott B. Teasdale

**Affiliations:** 1 Discipline of Psychiatry and Mental Health, School of Clinical Medicine, Faculty of Medicine and Health, UNSW, Sydney, NSW Australia; 2 Mindgardens Neuroscience Network, Randwick, NSW Australia; 3 South Eastern Sydney Local Health District, Sydney, NSW Australia; 4 Brain and Mind Centre, The University of Sydney, Sydney, NSW Australia; 5 Sydney School of Medicine (Central Clinical School), The University of Sydney, Sydney, NSW Australia; 6 Australian Research Council (ARC) Centre of Excellence for Children and Families over the Life Course, Sydney, NSW Australia; 7 Schizophrenia Research Unit, Ingham Institute for Applied Medical Research, UNSW Sydney, Liverpool, New South Wales, NSW 2052 Australia

**Keywords:** Co-design, Psychiatry, Health inequities, Qualitative research, Food security

## Abstract

**Aims:**

People living with a severe mental illness are more likely to experience food insecurity than people without mental illness. Food insecurity is linked to a range of negative socioeconomic and health outcomes. This study qualitatively explored causes and experiences of food insecurity for people living with a severe mental illness and used these findings to co-design a pilot intervention.

**Methods:**

Two workshops and seven focus groups were conducted as part of a co-design process involving people living with severe mental illness experiencing food insecurity (*n* = 9) and mental healthcare professionals, including peer workers (*n* = 11). These co-design sessions involved qualitative exploration of causes, experiences and impacts of food insecurity, and the use of implementation science tools to develop a pilot intervention. Notes and transcriptions were analysed using reflexive thematic analysis and directed content analysis for different co-design stages.

**Results:**

Two key causes of food insecurity emerged: a lack of access and a lack of education; these were exacerbated by multiple compounding factors. Food insecurity was also perceived as having systemic, bidirectional effects across mental and physical health. Incorporating community and peer support into solutions was seen as key, with ambivalence around financial support. A pilot intervention model targeting education issues through a peer-supported skill-building programme and access issues through a financial supplement was developed and rated highly in terms of feasibility, appropriateness and acceptability.

**Conclusions:**

Our findings reveal multifactorial perceived causes, experiences and impacts of food insecurity for people living with severe mental illness. To our knowledge, this is the first study to explore a lack of education as a core cause for this population group and to co-design a specialised intervention. The co-design methodology is a promising approach to developing practical and useful solutions to problems, using knowledge gained from the people involved in their implementation.

**Supplementary Information:**

The online version contains supplementary material available at https://doi.org/10.1007/s00127-026-03082-8.

## Introduction

Food security has been defined as the consistent and assured access to, and availability of, safe sufficient food to support nutritional adequacy and a healthy life, and deemed a human right by the United Nations [1]. Contemporary definitions further emphasise the importance of culturally appropriate foods, sustainability, and the stability of access over time, acknowledging that food insecurity also encompasses its acceptability and agency in obtaining it [2–4]. The Food and Agriculture Organization of the United Nations estimated that 8.9% of people worldwide experienced food insecurity (FI) in 2020, issuing an international call to action [3]. Yet FI has continued to rise, reaching 9.8% globally in 2021, with conflict, climate change and economic shocks contributing to widening inequalities [5]. In Australia, a nation-wide survey conducted in 2022 estimated that a third of households (33%) experienced moderate or severe FI, meaning that, at worst, households went entire days without eating [6]. This is concerning given the multiple detrimental physical and mental health outcomes of FI [7, 8].

Many complex factors influence the degree of food security. At an individual or household level, these may include financial constraints, storage and cooking facilities, cooking skills, and nutrition knowledge. Furthermore, food supply is affected by location, food availability, price, quality, and variety [9, 10].

In high-income societies such as Australia, socially disadvantaged groups experience FI more frequently than the general population [11]. When facing disadvantage, food can be perceived as a discretionary expense relative to other needs [11]. This commonly coincides with the over-consumption of non-nutritious foods, and lower intakes of core foods such as fruits and vegetables [7]. Consequently, this increases the risk for obesity and diet-related disease [12].

People with lived experience (PWLE) of severe mental illness commonly experience social disadvantage and health inequalities [13, 14]. A 2023 global meta-analysis found that the prevalence of FI in people with major depression, bipolar disorder, and schizophrenia and related psychoses was 2.7 times higher than the general population. The odds of FI for people living with severe mental illness were also higher in high/high-middle income countries compared to low/low-middle income countries, likely due to the high FI rates in the general population of low/low-middle income countries. FI was linked to a range of socioeconomic factors and detrimental health outcomes such as greater reliance on support networks, poorer financial status, and suboptimal lifestyle factors (nutrition, physical activity, and tobacco) [12]. Despite this, there is a lack of complementary research investigating the causes of FI for PWLE, their experiences, and how to support access to sufficient food of high nutritional quality [15–17].

This study had multiple staggered aims [18] including (a) understand how PWLE and healthcare professionals (HCPs) in Australia perceive the causes of FI, (b) understand the experience and impacts of FI, and (c) co-design a program to reduce FI, to implement a pilot intervention within mental health services.

## Methods

### Design

This mixed-methods explanatory sequential co-design process was conducted in a community mental health service in South Eastern Sydney Local Health District (SESLHD), Australia between March 2023 and February 2024. Consistent with previous research in intervention development for mental health services [19], two frameworks provided an evidence-based structure for the co-design process: (1) the 2021 Medical Research Council framework for developing complex interventions [20], including developing, testing the feasibility, evaluating, and implementing the intervention and (2) a co-production framework [18], that seeks lived experience leadership and input from the outset. In accord with this latter framework, a lived experience co-author (COD) was engaged throughout the project. However, while the lived experience perspective was privileged and decision-making shared, conventional researchers [21] outnumbered PWLE, thus this study may be considered ‘working towards’ true co-production in line with Bellingham and colleagues [22].

Ethical approval was received from the SESLHD Human Research Ethics Committee (2022/ETH02327). Reporting adheres to the standards for qualitative research and guidance for intervention development studies in health research [23, 24] *Supplementary Files 1 and 2.*

### Sample

There were two distinct participant groups: (i) PWLE, and (ii) HCPs. Inclusion criteria for PWLE were: (a) people living with a diagnosis of a severe mental illness, defined as schizophrenia and related psychoses, and bipolar disorder (ICD-10 codes F20 to F31) [25], (b) aged ≥ 18 years old, (c) receiving treatment via mental health services within the SESLHD catchment, (d) community-dwelling, (e) having experienced moderate or severe FI in the previous 12-months determined by the Household FI Access Scale (HFIAS), an 18-item structured assessment [26], (f) comfortable communicating in English, including through a professional or non-professional (e.g., support worker) interpreter, and (g) able to provide consent, determined by their clinical team.

Inclusion criteria for HCPs were: (a) Mental health clinicians or peer workers in SESLHD community mental health services, and (b) willing to provide consent.

### Recruitment

PWLE were recruited from a community mental health centre in SESLHD. HCPs provided expression of interest forms to potential participants, taking into consideration the inclusion criteria except for FI. Interested people then contacted the research team (ST or OAY) who met with the person via telephone or in-person, and screened for FI via the HFIAS [26]. Those who met inclusion criteria completed an informed consent procedure with the researcher, and potential participants took at least 24 h to consider their consent.

HCPs were recruited from the same adult and youth community mental health centres in SESLHD. A maximum variation approach was taken to sampling for this group in relation to disciplinary background (i.e., nursing) and role (i.e., clinical team leader). HCPs were recruited through email and verbal invitations during meetings and were given equivalent information and minimum timeframes to consider participation as the PWLE group.

We planned to recruit *N* = 10 PWLE and *N* = 8 HCPs and to cease recruitment once this sample size was reached. This was to establish a relatively equal power dynamic amongst groups, to account for expected drop-out across sessions, and guidance for workshops and focus groups [27].

### Co-design process

Co-design is the bringing together of people with experience of the health system to design processes and programs based on their lived experiences [28]. We employed a combined process of both partnership through co-design and an implementation-based approach to guide intervention appropriateness, acceptability and feasibility [29]. Two 6-hour workshops and seven 1.5-hour focus groups were conducted. An iterative knowledge translation process was applied, so that ideas generated within earlier stages were refined and elaborated upon by participants in later stages following a methodology of discovery, evaluation and prototyping phases [30, 31]. Creation of the conditions for co-design was guided by McKercher’s principles and mindsets, which place at the core of the process the values of recovery-oriented and trauma-informed care [32].

### Phase 1: Co-design workshops

Co-design workshops were held in a university seminar room. Workshops were co-facilitated by a translational researcher with a professional background as a psychologist (AM), and a lived experience lead with experience working as a peer worker (COD or KB). Two additional researchers with backgrounds in dietetics (ST) and psychology (OAY) provided onsite support. An outline of the purposes and activities of each workshop are given in Box 1.

Box 1. Purpose and activities of workshops **Workshop 1**

The background, aim and scope of the project were discussed, and the concept of co-design was introduced, focusing on capacity-building and power dynamics [33]. Group activities included defining FI, understanding the causes, experiences and impacts on mental and physical wellbeing of FI, a World Café [34] exploring potential solutions of a food pantry, peer-delivered life skills program, or financial supplement, brainstorming blank canvas solutions, and discussion of mental health service screening for FI.

The three seed solutions were generated by the topic expert (ST). Group activities involved small group discussion before feeding back to the whole workshop, and participant backgrounds were intentionally mixed in these small groups to enrich discussions [35]. Following Workshop 1, researchers reviewed all potential solutions for feasibility and appropriateness, narrowing the list to five potential solutions. These were taken forward to Workshop 2.

**Workshop 2**

Workshop 2 included a re-orientation to content covered in Workshop 1. Group activities included further exploration of the five potential solutions against the ‘5 W’s and H’ approach: *who*,* what*,* where*,* when*,* why* and *how* [36]. Participants then individually prioritised the five solutions by ranking ideas on a paper hand-out. The three solutions rated as the highest priority were refined into an intervention model which were then rated individually for their feasibility, acceptability and appropriateness (see data collection and data analysis). Finally, these aggregated ratings were fed back to the workshop and a collective vote was held on which two models could best be combined.

### Phase 2: Focus groups

Focus groups, which had an aim of user-testing the intervention prototype developed in workshops, were held at the adult community mental health centre and facilitated by ST and OAY, audio-recorded using Microsoft Teams. Participants were offered the opportunity to participate in mixed or closed (e.g., only PWLE) groups. The focus groups involved a guided walk-through of an intervention model synthesised from Workshop 2 as a form of member-checking, and exploration of participants’ experiences of co-design [37]. The model was displayed as an A1 print-out which could be written or drawn on, and focus groups were audio recorded. Questions related to the model were derived from the Consolidated Framework for Implementation Research (CFIR) [38] and prompts regarding comparison to alternative interventions were informed by local food security support directories. Co-design questions were adapted from Siig Pallesen and colleagues [39]. Question guides are given in *Supplementary File 3.*

### Data collection

A dedicated facilitator (OAY) took detailed notes throughout the co-design workshops and focus groups. Butcher’s paper was used as a canvas to record participants’ thoughts during workshop activities. Individual notebooks were also provided if participants felt more comfortable contributing this way. Field notes, all physical materials used as part of the co-design, and audio recordings were collected as artefacts for qualitative analysis. Field notes and physical materials were transcribed verbatim in Microsoft Word and automatic transcriptions generated by Microsoft Teams were reviewed for accuracy against the audio recording.

To understand preliminary implementation factors, participants’ perceptions of the acceptability, appropriateness, and feasibility of potential service models refined through co-design in Workshop 2 were measured using the Acceptability of Intervention (AIM), Intervention Appropriateness (IAM), and Feasibility of Intervention Measures (FIM) questionnaires [40]. Participants rated their level of agreement with a total of 12 statements using 5-point Likert scales, anchored at “1 = Strongly disagree” and “5 = Strongly agree”.

The survey at the end of workshops explored participants’ experiences of the co-design process and collected demographic details. Questions regarding co-design were adapted from McKercher [32]. Demographics collected for PWLE included: age, gender, cultural background, proficiency in spoken English, main language spoken at home, employment and housing status, and duration of mental health service use. Demographics collected for HCPs included: age, gender, profession, and duration of employment in mental health services. Design and interpretation were informed by the Australian Standards [41].

### Data analysis

Descriptive statistics were calculated for demographic data. Mean values were calculated for each subscale of the AIM/IAM/FIM measures using SPSS Statistics (IBM Corp., Version 27, Armonk, NY), with higher means indicating greater acceptability, appropriateness, or feasibility.

Reflexive thematic analysis was employed for analysis of artefacts from Workshop 1 adopting a constructionist epistemology [42, 43]. Following familiarisation during transcription, inductive semantic and latent line-by-line coding was conducted independently by OAY, COD and ST to develop a thematic framework. The framework was iteratively reviewed with AM and divergence in coding was resolved through discussion at research meetings. Following three iterations, OAY coded the artefacts in NVivo 14 Software^®^.

Directed content analysis was employed for artefacts from Workshop 2 and focus groups [44, 45]. For Workshop 2 a thematic framework was developed by COD and OAY in a similar process to that described above, drawing additionally on themes from Workshop 1. For focus groups a hybrid inductive/deductive framework was created. Constructs comprising acceptability were drawn from the theoretical framework of Sekhon and colleagues [46] Feasibility and appropriateness were framed in language drawn from the co-design process and informed by Bowen and colleagues [47] and the CFIR [38]. COD and OAY each coded 50% of the artefacts independently in NVivo 14 Software^®^. All authors discussed and reviewed the final themes.

## Results

### Participants

**Fig. 1:**
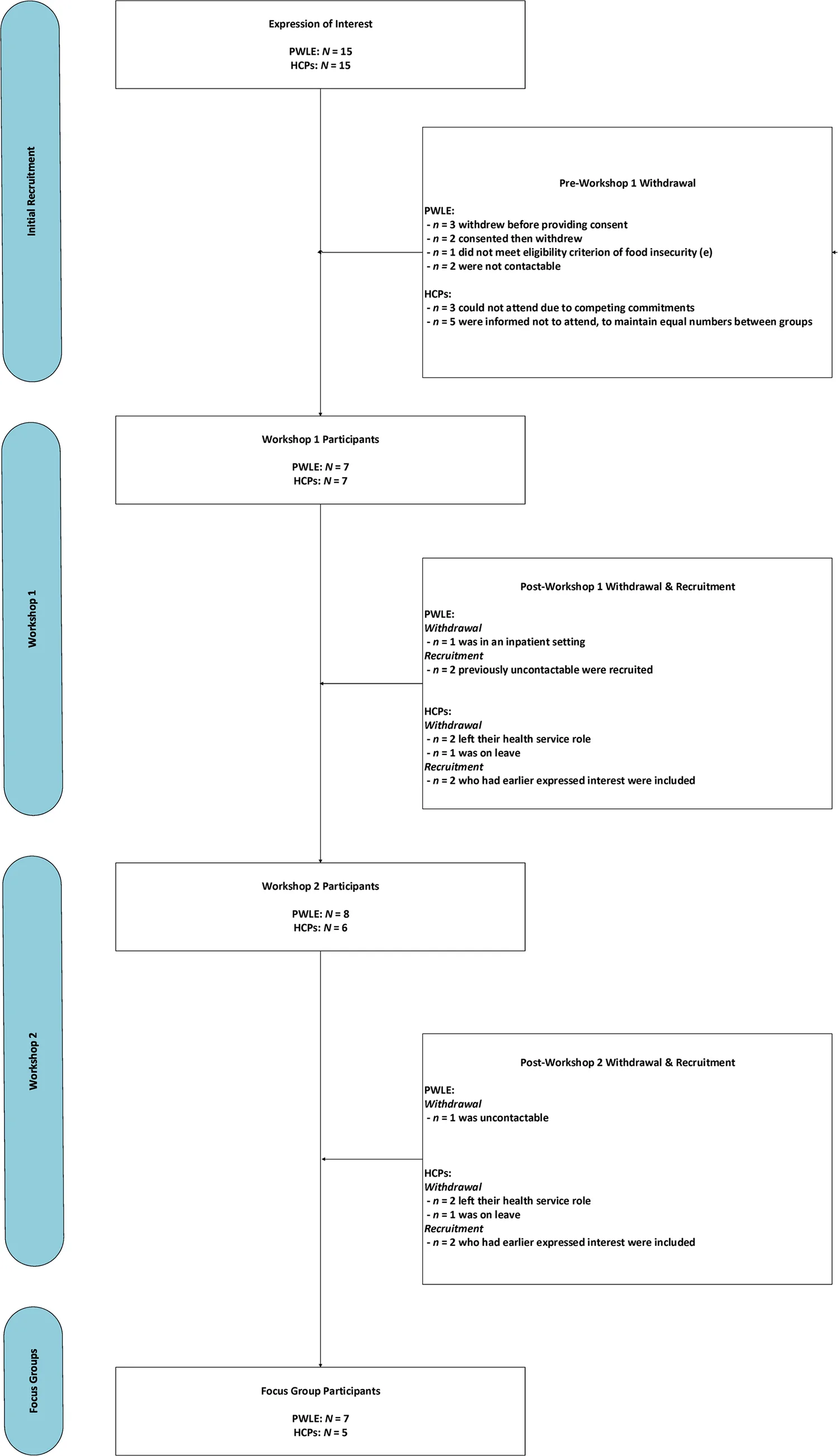
Participant flow

A total of 15 PWLE expressed interest in the study and nine participated over the course of the co-design process (*n* = 5 attended all stages). Fifteen HCPs expressed interest in participating and 11 participated (*n* = 3 attended all stages). Figure 1 details the participant flow and Table 1 displays demographic details for both participant groups.

**Table 1:** Demographics of participants

Variable	Community Participants (*N* = 9)		Healthcare Professionals (*N* = 11)	
	*N*/Mean	%/SD	*N*/Mean	%/SD
Gender^1^				
Male-identifying	2	22.2%	0	0%
Female-identifying	4	44.4%	10	90.9%
Non-binary	2	22.2%	1	9.1%
Prefer not to say	1	11.2%	0	0%
Duration of experience with mental health service (years)^1^				
< 2 years	0	0%	1	9.1%
≥ 2 years	9	100%	10	90.9%
Previous codesign experience				
Yes	2	22.2%	9	81.8%
No	7	77.8%	2	18.2%
Age (years)^2^	46.0	13.3	N/A	N/A
Cultural background^3^			N/A	N/A
Australian	6	66.7%		
New Zealander	2	22.2%		
Aboriginal	1	11.2%		
Maori	1	11.2%		
Irish	1	11.2%		
Spanish	1	11.2%		
Filipino	1	11.2%		
Singaporean	1	11.2%		
Greek	1	11.2%		
Accommodation				
Government rent	9	100%	N/A	N/A
Employment status				
Unemployed	9	100%	N/A	N/A
Spoken English level				
Very good	7	77.8%	N/A	N/A
Good	2	22.2%	N/A	N/A
Disciplinary background	N/R	N/R		
Nurse			3	41.3%
Social worker			2	18.2%
Occupational therapist			2	18.2%
Peer worker			2	18.2%
Dietitian			2	18.2%

### Workshop 1

#### Causes and experiences of FI

**Fig. 2:**
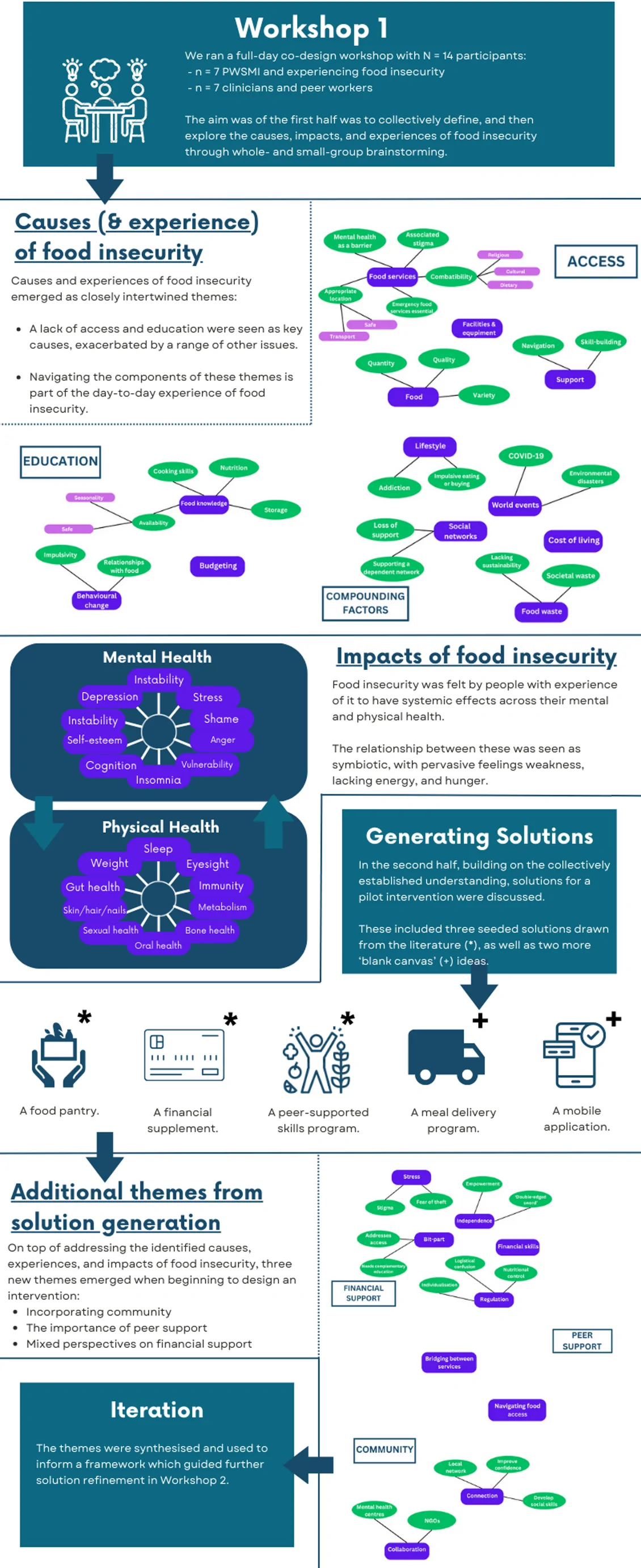
Workshop 1: Causes, experiences and impacts of food insecurity, and potential solutions

Three themes emerged related to the causes and experiences of FI: (i) access, (ii) education, and (iii) additional compounding factors. Access and education were framed as core causes of FI and were exacerbated by a range of compounding factors, as illustrated in Fig. 2.

### Theme 1: Access

Access issues involved multiple, overlapping barriers. Participants described challenges engaging with food relief services that were compatible with their religious, cultural or dietary needs, and that were conveniently located, whilst also facing uncertainty about ‘where the next meal is coming from’: “*People who are halal or kosher*,* they can’t accept donated food*”. These difficulties were compounded by mental ill-health and stigma associated with using these services: “*You’re relying on services… if that person doesn’t come one week*,* what happens then?*”. Participants also reported lacking adequate facilities and equipment for cooking and storage: “*Not having a fridge*”. Others described limited support with navigating food services – often necessary when managing periods of mental ill-health - or developing cooking skills: “*Not having the skills or resources to prepare food that you have*”. Finally, some expressed difficulty obtaining sufficient food of good quality and variety: “*I skip meals as there is a lack of variety*”.

### Theme 2: Education and skills

Issues with education centred on knowledge, behaviour change and priorities. Participants discussed gaps in understanding of food availability, nutritional value, and cooking and storage skills: “*Can you use what you buy?*”. They also described difficulties in changing behaviours related to heightened impulsivity or lack of motivation due to mental ill-health, and relationships with food: “*Need to want to eat ‘nutritious’ foods*”. Managing finances was another challenge with some participants reporting food becomes a lower priority when finances are limited: “*Food is the least important on my budget.*”

### Theme 3: Compounding factors

Access and educational issues were exacerbated by broader socio-economic and ecological pressures. Participants described the difficulties associated with a loss of social support or through the strain of supporting other people or pets: *“I sometimes don’t have food for myself but have food for my dog*”. Some participants also reported issues related to co-occurring substance use: “*Addiction expenses means less money for food*”. Further, participants highlighted an increased cost-of-living having dire consequences, particularly in relation to health: “*Having to choose between paying for food or medication*”. Participants also identified macro-level factors of world events such as climate disasters or pandemics: *“[The] pandemic made us more aware of vulnerable groups*”. Finally, participants called to attention the large amounts of food waste in the world: *“[Corporate] functions… it all goes in the bin*”.

### Impacts

FI was perceived as having a profound and interconnected impact on both physical and mental health.

### Theme 1: Mental health

This connection was highlighted through general feelings of “*weakness*”, “*lacking energy*” and “*lacking motivation to move/exercise/socialise*”. The effects described on physical health were systemic and far-ranging, as illustrated in Fig. 2, including “*teeth decay*”, “*weight loss*”, “*fertility issues*”, being “*prone to illness*”, and “*side-effects*” of medication, and impacts on a person’s ability and desire to manage chronic conditions. Participants expressed feelings of apathy and resignation about these impacts, making comments such as “*I have diabetes but I don’t care about it*”.

### Theme 2: Mental health

The impact on mental health was centred predominantly on the toll of “*instability*” and “*vulnerability*”, feeling “*suffocated*” and “*helpless*” with chronic stress. The interplay of these mental and physical effects was epitomised by one participant stating that “*[You] can’t sleep if you’re stomach’s croaking from lack of food – it’s very distressing*”. Other impacts included perceived impaired cognition, in terms of “*memory*” and “*concentration*”, as well as a range of other feelings including “*guilt*”, “*depression*”, “*shame*”, and “*jealousy*”. Further, FI leads to compensatory actions, exacerbating these feelings, with one participant sharing that they “*had to steal – I did it the other day!*”.

### Solution design

Twenty-four ideas emerged during ‘blank slate’ brainstorming. After assessing feasibility, appropriateness and acceptability of each of the 24 ideas, two potential solutions were added to the three solutions drawn from the literature. Descriptions of each of these potential solutions are presented in Box 2.

Box 2. Seeded and ‘blank slate’ solutions from Workshop 1 **Seeded solutions**:

1. A peer-supported skill-building program would aim to “*increase skills and qualifications”* and could include “*travel training”*,* “shopping tours outside of [supermarket] hours”*, a “*community kitchen teaching people to cook”* and utilise existing “*[National Disability Insurance Scheme] support for cooking and shopping [and] meal delivery”.*
2. A financial supplement, also referred to as a Healthy Food Incentive, could be implemented through a variety of different ways, such as “*protected income for co-ops”*, a “*discount card”*,* “protected income card”*, or “*coupons*” with the aim that *“everyone has the same basics”*.
3. The way in which a food bank could be carried out was also developed in multiple directions. These included: a pantry or soup kitchen at the community mental health centre which *“incorporate[s] volunteers [for] (prep/upskilling*)”, “*food hamper[s] on discharge”*, a “*BBQ regular time once a month”*, or a “*Mobile food pantry”* which also provided “*extras (e.g.*,* blankets*,* toilet bags)”*.

**‘Blank slate’ solutions**:

4. A mobile phone application with “*affordable recipes that are nutritious/tasty”* and/or information on services for “*where to get a hot meal/partner with them”.*
5. A meal delivery program, either providing access to “*frozen meals [for] free”* or in which “*cafes/restaurants deliver a meal”* and are reimbursed through a government scheme.

Across these brainstormed solutions, three design-centred themes emerged: (i) the pros and cons of financial support, (ii) the need for peer support, and (iii) the need for community.

### Theme 1: Financial support

Financial support was viewed ambivalently and described as a *“double-edged sword of having money freed up*,* [which] can be either positive or negative- either for vet bills*,* clothing or drugs or cigarettes*”. This meant that it was considered a bit-part solution on its own, as “*It’s not sustainable in the long-term – what’s it teaching you*?”. This solution also does not account for the stress and stigma associated with attending a supermarket, with participants commenting that “*each time you go to the supermarket you need a tough face*,* a lot of stigma*,* people might steal [vouchers/a card]*”. Concerns were also raised about its regulation in terms of logistical confusion, such as “*Does it go in Centrelink [government support] benefits?*” and nutritional control, raising issues over “*who decides [what] is nutritious*?”. The benefits of individualisation and autonomy were contrasted with a need to consider capacity-building of financial skills, asserting that solution efforts would “*[need to assist with] money-management*”.

### Theme 2: Peer support

The inclusion of peer support into a solution model consistently emerged, to aid navigation in accessing food services, such as to *“go out [with the] person into the community 1:1 > then [the] person can feel confident going in the future to get to services in the community”* and in ‘bridging’ between disability support, charities and the mental health service, with participants observing that these are “*currently all separated > good to be integrated*”.

### Theme 3: Community

Building community into a solution was viewed as key, to create collaboration between different kinds of services (e.g., providing clothing, cooking equipment, technology). Participants perceived that “*if we strengthen those relationships*,* it can go beyond the provision of food to more for the community*” and addressing social dimensions of the issue to create connection, stating that*“[we need to generate feelings of] acceptance and social connection*”.

### Workshop 2

Of the five solutions explored in depth, the Peer Support Program, Financial Supplement and Food Pantry were prioritised through the individual ranking activity. Further development occurred through small group activities, resulting in prototype models of care (*Supplementary File 4)*. Directed content analysis conducted on these models is displayed in Table 2.

**Table 2:**
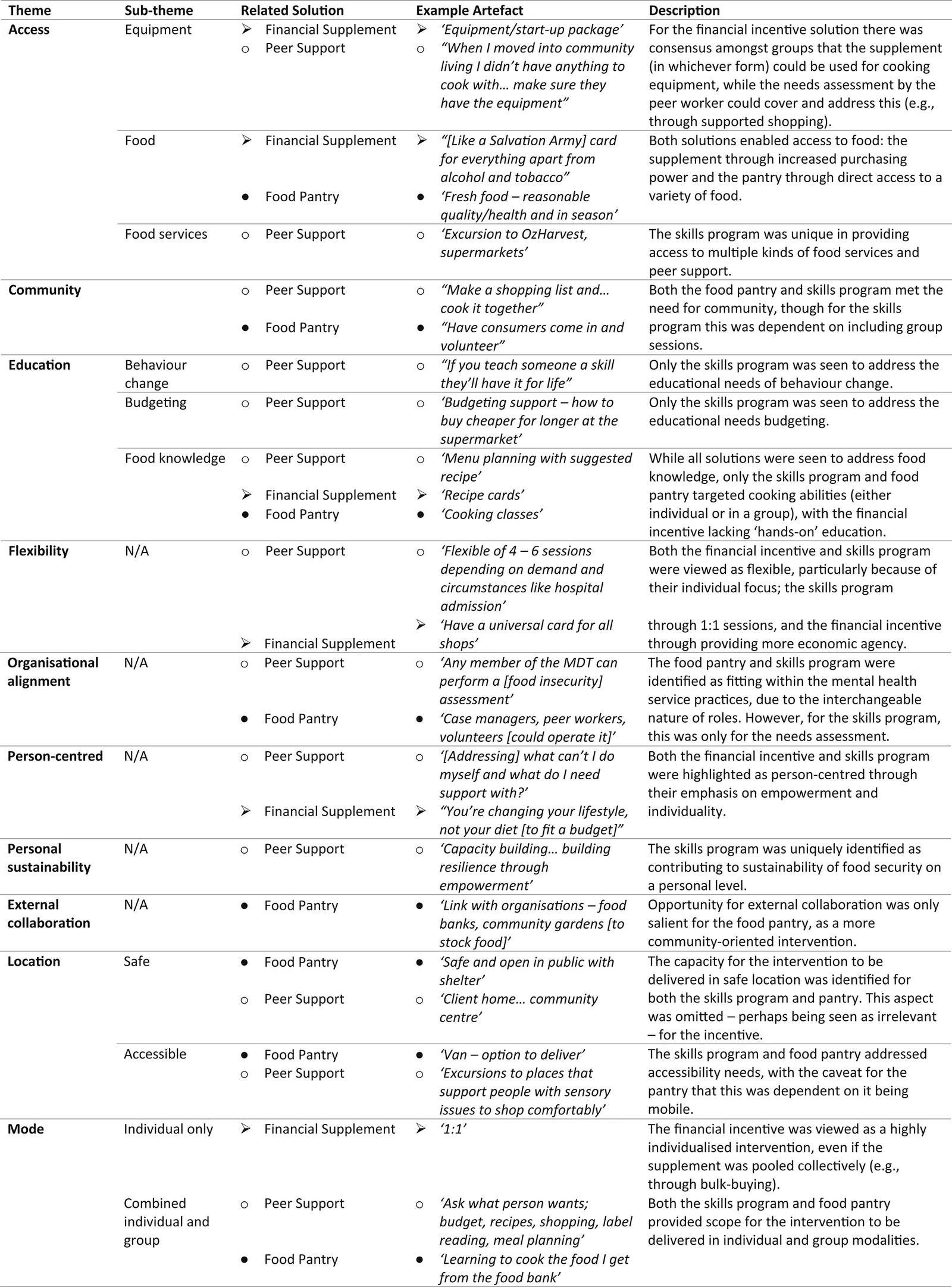
Directed content analysis of Peer Support Program, Financial Supplement and Food Pantry potential solutions

N/A = Not applicable

Participants rated the Peer Support Program as the most acceptable (M = 4.36 ± 0.55), followed by the Financial Supplement (M = 4.31 ± 0.66) and the Food Pantry (M = 4.17 ± 0.90). The Peer Support Program was also rated the most appropriate (M = 4.27 ± 0.57), marginally higher than the Financial Supplement (M = 4.22 ± 0.86) and the Food Pantry (M = 4.00 ± 0.60). The Financial Supplement was the highest rated solution for the outcome of feasibility (M = 3.92 ± 0.79), compared to the Peer Support Program (M = 3.84 ± 0.80) and the Food Pantry (M = 3.72 ± 0.48). In collective voting, the financial supplement and peer-supported skill-building were viewed as the best two models to combine.

### Focus groups

**Fig. 3:**
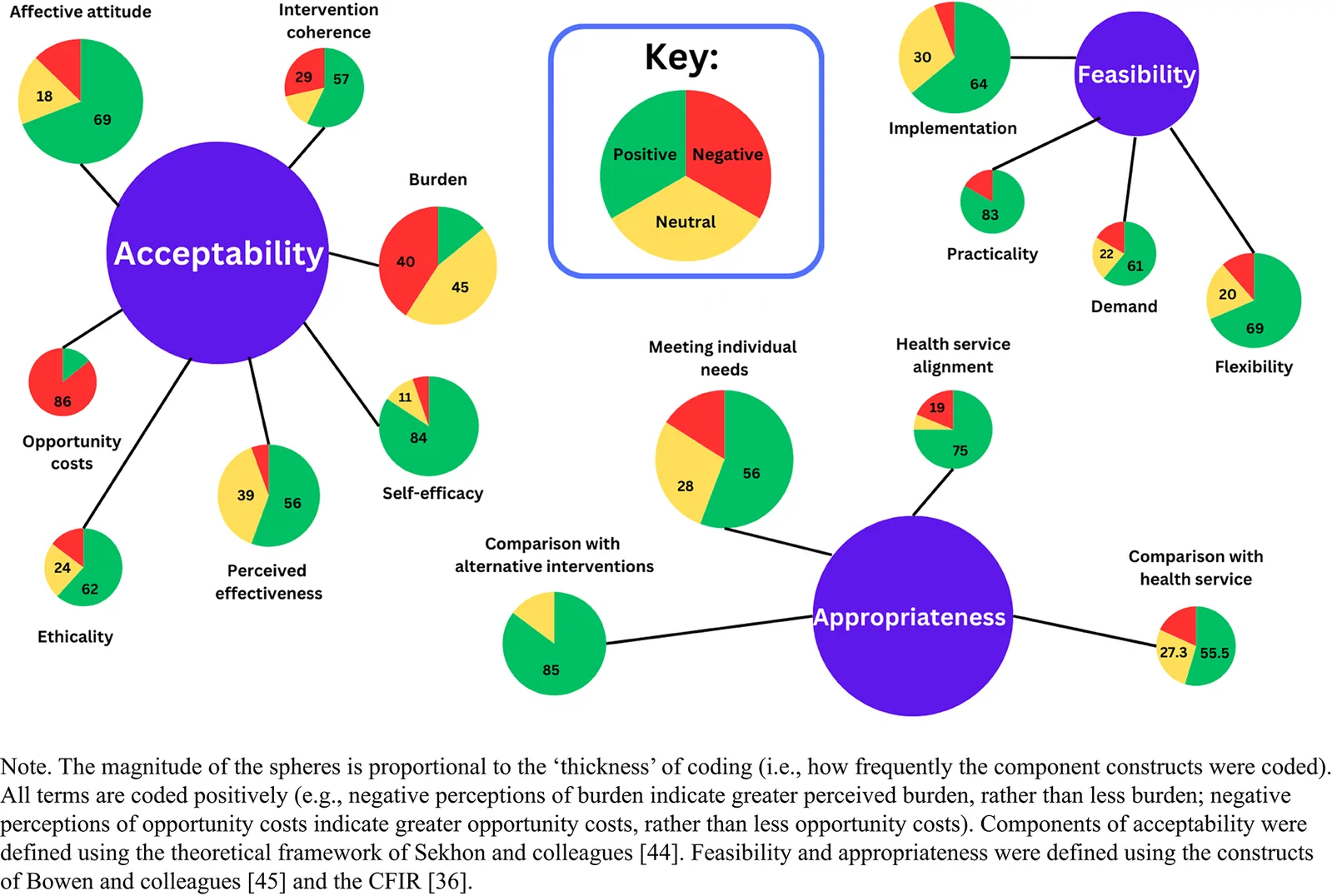
Directed content analysis of positive, negative, and neutral perceptions of the pilot intervention’s acceptability, appropriateness, and feasibility.

Seven focus groups were conducted in total, one of which was closed (available only to PWLE) and comprised between one and three participants. Box 3 outlines the pilot intervention model presented to participants, synthesised from Workshop 2 findings, based on Michie and colleagues’ guidance [48]. Figure 3 displays directed content analysis of perceptions of the pilot intervention.

Box 3. Prototype pilot intervention presented during focus groups **Characteristics of the recipients**:

Adults living with a diagnosis of a severe mental illness in the community, who are moderate or severely food insecure, are below the poverty line, and engaged with the community mental health service.

**Characteristics of those delivering the intervention**:

A specialist food insecurity peer support worker and an occupational therapist.

**Recruitment**:

Screening for eligibility conducted by healthcare professionals, based on recipients’ interest in support. The peer support worker and occupational therapist complete a comprehensive assessment with the recipient, covering: budgeting, meal planning, food safety, food access, and cooking skills, as well as the need for equipment for preparation and storage.

**Intervention content and structure**:

*Peer Support Program*: 1:1 sessions with the peer support worker, with content guided by the comprehensive assessment and the recipients’ goals.

*Financial Supplement*: Weekly $100 supplement via a cashless debit card which can be used at a major supermarket chain, with restrictions on alcohol and tobacco.

Adjunct*: Existing cooking and nutrition groups at the community mental health service.

**Setting**:

In the recipients’ home or at the community mental health service.

**Mode of delivery**:

Depending on the preference of the recipient: a mix of face-to-face or online sessions.

**Intensity and duration**:

*Peer Support Program*: 6 sessions used flexibly over 12 weeks.

*Financial Supplement*: Weekly over 12 weeks.

*Adjunct*: Weekly over 6 weeks.

Note* The adjunct cooking groups are part of the Keeping the Body in Mind program [49].

### Acceptability

The model was viewed broadly positively in terms of acceptability. In particular, the combined targeting of access issues through the financial supplement and educational issues through a skills program was viewed as a complementary and effective approach: “*People aren’t gonna change their diet [just] because they’ve got a free card.*” (PWLE).

Additionally, restricting the financial supplement solely for food and equipment and the requirement to engage in the skills program to receive the supplement were also perceived as positives: “*There’s [still] responsibility*,* agency and self-determination in that.*” (Peer Worker).

Though not seen as outweighing the potential benefit of the intervention, participants also highlighted potential opportunity costs of engaging with the intervention. For example, if the financial supplement was only available at a specific supermarket, causing people to change their routine or posing additional barriers: “*I only have Woolies [supermarket] near me*,* I’d have to get someone to drive me to the Coles [supermarket].*” (PWLE).

Lessening possible burdens of participation was the key concern for improving the acceptability of the pilot model, including making sure that in-home sessions are unintrusive and reducing the onus on the participant during an assessment stage: “*Even though $100 a week is good*,* I kind of don’t want people in my personal business – you know what I mean?*” (PWLE).

### Appropriateness

The model was perceived as relevant and well-fitting, and as an improvement on existing interventions within the health system or alternatives from organisations providing food security support: “*It’s a massive gap and something needs to fill it. There might be the occasional hit and miss thing*,* but in general*,* yeah*,* nothing like this exists.*” (PWLE).

The adaptability of the model, with the option for different locations and timeframes for sessions and individualised assessment was seen as a key strength, allowing for different health system structures and individual needs. However, some participants noted that the intensity of support may be too little for some people: “*I think six sessions and no [more] support won’t build that scaffolding… they could too easily drop out in a week*” (HCP).

### Feasibility

Participants described the model as practicable and suitable, emphasising high demand and the flexibility of how support could be used for building different skills: “*If they don’t wanna do budgeting*,* then they don’t have to*” (PWLE). While it was viewed as broadly implementable, its scalability across different health districts was seen as a challenge: “*How we’re gonna tackle that hurdle of educating and building up that skill in clinicians so that they can actually be aware this person might be experiencing these issues and might find this program useful*” (PWLE).

## Discussion

Our findings reveal wide-ranging and unique perceived causes, experiences and impacts of FI for PWLE. The plurality of these highlighted the inadequacy of solutions designed to address FI for the general population and the need for individualised support. The co-design process undertaken, is, to our knowledge, the first within this area and demonstrates a promising approach to developing solutions which are feasible, acceptable and appropriate.

### Causes, experiences, and impacts of food insecurity

On top of typical causes of FI in the general population, such as issues with transport, the cost-of-living, and stigma [10], PWLE face additional challenges, such as periods of mental ill-health as a barrier to accessing food and potentially managing addictive or impulsive behaviours. Even within traditional causes, participants expressed a perceived augmented stigma of FI due to mental illness or pressure of cost-of-living increases on the price of medication [16]. That FI is one of many competing priorities, when considering medications to manage illness, addictions, or other basic needs such as shelter, underscores the multidimensionality of causes and experiences.

Our results emphasise a lack of education around nutrition, cooking and food storage, and a shortage of cooking facilities and equipment – causes which have not been examined in comparable qualitative studies [16, 17]. The appropriateness of food security interventions that focus on food skills in the general population have been subject to criticism for failure to address inadequate financial resources and systemic inequities [50–52]. However, the voices in our co-design were clear: targeting issues of access alone is not enough. Without educational support, interventions for PWLE may be ineffective in ameliorating inequity. A tandem approach is needed.

We found that the impacts of FI were perceived as pervasive across the whole span of health and wellbeing. This complements the existing quantitative literature reporting higher levels of psychological distress, obesity, and sedentary behaviour [12] and echoes findings of Giles and colleagues [16]. In their peer-led interviews with PWLE experiencing food insecurity, participants discussed a vicious cycle wherein FI negatively impacts mental and physical health, which in turn increases FI [16].

### Co-designed solutions

As emerged in our workshops, the most common existing intervention model of food banks has been criticised for doing little to reduce stigma [53], offering poor nutritional quality [54], and lacking accessibility [16]. The co-designed pilot intervention was identified as unique and with advantages over existing interventions such as food banks, as well as identifying key considerations for implementation of the intervention. As this was not based on an existing intervention model, considerations for resource allocation and infrastructure are needed to ensure it is viable.

### Strengths and limitations

Limitations of our study include lack of culturally and linguistically diverse and First Nations participants, as these groups face elevated risk for experiencing FI [7, 55, 56] and an inability to generalise to other (e.g., regional) contexts. Additionally, there is the potential that participant responses were based on perceived social desirability, though multiple options for participating were provided, and there was a lack of perspectives from policymakers and food support service workers.

A key strength of the study is the rigorous approach to developing a targeted solution. Previous FI qualitative research with PWLE has only evaluated existing interventions, such as food banks, or methods of increasing advocacy for food justice [16, 17, 57]. Co-design with PWLE and HCPs – including peer support workers – [15, 58, 59] is known to improve implementation outcomes [28, 38, 60] and respects the ethical need for those who are directly impacted by research to actively contribute [61]. Further, co-facilitation of co-design by PWLE enables a certain level of comfort for participants when discussing mental health and FI [16]. The co-production of the research process recognises that the knowledge and expertise of PWLE is essential for creating quality services and relationship building to engender benefits beyond the project [18]. The mixed-methods design and incorporation of implementation science theory regarding acceptability, appropriateness and feasibility [62] also aids translation of our findings, and the iterative approach enabled a methodology responsive to feedback, to increase engagement.

### Future directions

Food security is a basic need that is not being met for PWLE. This may be considered a root issue [12] driving unacceptable inequity of physical health disparities prevalent in this population [14] and undermining promising evidence that dietary interventions can help improve physical health. Our study reaffirms the need for including assessment of FI as part of routine clinical care [12]. A brief, trauma-informed method for mental health clinicians to routinely ask PWLE about food security needs to be established and validated.

The new intervention model also needs complementary policy reform [63] addressing the structural determinants identified (e.g., cost of living increases) to prevent a ‘sticking plaster’ solution isolated to one high-resource setting [16]. The themes which emerged from Workshop 1 may serve as a framework for scaffolding such reform. The content analysis of Workshop 2 may be used as a tool for services self-evaluating or developing interventions in other contexts.

## Conclusion

Our co-design identified multiple perceived causes, experiences and impacts of FI for PWLE, adding to scarce existing qualitative literature in this area. Importantly, diverging from other studies, issues with both access and education emerged as key for this population group. This study also presents a novel pilot intervention model for implementation, incorporating a flexible peer-supported skill-building program and financial assistance to directly address education and access.

## Supplementary Information

Below is the link to the electronic supplementary material.

Supplementary Material 1

Supplementary Material 2

Supplementary Material 3

Supplementary Material 4

Supplementary Material 5

## Data Availability

Data sharing is available upon reasonable request and approval from the South Eastern Sydney Local Health District Human Research Ethics Executive Committee.

## Declarations

### Permission to reproduce material from other sources

No material from other sources has been reproduced.

## References

[1] FAO (2005) The Right to Food: Voluntary guidelines to support the progressive realization of the right to adequate food in the context of national food security

[2] FAO (2015) Voices of the Hungry: Measuring Food Insecurity Through the Food Insecurity Experience Scale (FIES)

[3] FAO, et al et al (2020) The State of Food Security and Nutrition in the World 2020. Transforming food systems for affordable healthy diets. Rome, FAO

[4] Committee on World Food Security (CFS) (2020) High Level Panel of Experts (HLPE) on Food Security and Nutrition Report #15: Food Security and Nutrition: Building a Global Narrative Towards 2030

[5] FAO, et al et al (2022) The State of Food Security and Nutrition in the World 2022. Repurposing food and agricultural policies to make healthy diets more affordable. Rome, FAO

[6] Miller K, Li E (2022) Foodbank Hunger Report 2022

[7] Lindberg R et al (2015) Food insecurity in Australia: Implications for general practitioners. Aus Fam Physician 44(11):859–862 26590630

[8] Pourmotabbed A et al (2020) Food insecurity and mental health: a systematic review and meta-analysis. Public Health Nutr 23(10):1778–1790 32174292 10.1017/S136898001900435X PMC10200655

[9] Booth S, Smith A (2001) Food security and poverty in Australia–challenges for dietitians. (Review paper). Australian J Nutr Dietetics 58:150

[10] Rosier K (2011) Food insecurity in Australia: What is it, who experiences it and how can child and family services support families experiencing it? Australian Institute of Family Studies, Editor

[11] Kleve S et al (2017) Are low-to-middle-income households experiencing food insecurity in Victoria, Australia? An examination of the Victorian Population Health Survey, 2006–2009. Aust J Prim Health 23(3):249–256 28076748 10.1071/PY16082

[12] Teasdale SB et al (2023) Prevalence of food insecurity in people with major depression, bipolar disorder, and schizophrenia and related psychoses: A systematic review and meta-analysis. Crit Rev Food Sci Nutr 63(20):4485–4502 34783286 10.1080/10408398.2021.2002806

[13] Morgan VA et al (2014) Psychosis prevalence and physical, metabolic and cognitive co-morbidity: data from the second Australian national survey of psychosis. Psychol Med 44(10):2163–2176 24365456 10.1017/S0033291713002973 PMC4045165

[14] Firth J et al (2019) The Lancet Psychiatry Commission: a blueprint for protecting physical health in people with mental illness. Lancet Psychiatry 6(8):675–712 31324560 10.1016/S2215-0366(19)30132-4

[15] Smith J et al (2023) Food insecurity and severe mental illness: understanding the hidden problem and how to ask about food access during routine healthcare. BJPsych Adv 29(3):204–212

[16] Giles EL et al (2024) Food insecurity in adults with severe mental illness living in Northern England: Peer research interview findings. Int J Ment Health Nurs 33(3):671–682 38059552 10.1111/inm.13270

[17] Myers N et al (2019) Coping with Food Insecurity Among African American in Public-Sector Mental Health Services: A Qualitative Study. Community Ment Health J 55(3):440–447 30825072 10.1007/s10597-019-00376-x

[18] Roper C, Grey F, Cadogan E (2018) Co-production: putting principles into practice in mental health contexts. Melbourne School of Health Sciences, University of Melbourne

[19] Milton A et al (2024) Co-Production of a Flexibly Delivered Relapse Prevention Tool to Support the Self-Management of Long-Term Mental Health Conditions: Co-Design and User Testing Study. JMIR Form Res 8:e49110 38393768 10.2196/49110 PMC10926903

[20] Skivington K et al (2021) A new framework for developing and evaluating complex interventions: update of Medical Research Council guidance. BMJ 374:pn2061 10.1136/bmj.n2061 PMC8482308 34593508

[21] Killackey E (2023) Lived, loved, laboured, and learned: experience in youth mental health research. Lancet Psychiatry 10(12):916–918 37625427 10.1016/S2215-0366(23)00270-5

[22] Bellingham B et al (2023) Co-design Kickstarter

[23] Duncan E et al (2020) Guidance for reporting intervention development studies in health research (GUIDED): an evidence-based consensus study. BMJ Open 10(4):e033516 32273313 10.1136/bmjopen-2019-033516 PMC7245409

[24] O’Brien BC et al (2014) Standards for Reporting Qualitative Research: A Synthesis of Recommendations. Acad Med, 89(9) 10.1097/ACM.0000000000000388 24979285

[25] World Health O (2004) ICD-10: international statistical classification of diseases and related health problems : tenth revision. World Health Organization: Geneva 3376487

[26] Coates J, Swindale A, Bilinksy P (2007) Household Food Insecurity Access Scale (HFIAS) for Measurement of Household Food Access: Indicator Guide (v. 3). FHI 360/FANTA: Washington, D.C

[27] Onwuegbuzie AJ, Leech NL (2007) A call for qualitative power analyses. Qual Quantity: Int J Methodol 41(1):105–121

[28] Slattery P, Saeri AK, Bragge P (2020) Research co-design in health: a rapid overview of reviews. Health Res Policy Syst 18(1):17 32046728 10.1186/s12961-020-0528-9 PMC7014755

[29] Proctor E et al (2011) Outcomes for implementation research: conceptual distinctions, measurement challenges, and research agenda. Adm Policy Ment Health 38(2):65–76 20957426 10.1007/s10488-010-0319-7 PMC3068522

[30] Milton AC et al (2021) Participatory Design of an Activities-Based Collective Mentoring Program in After-School Care Settings: Connect, Promote, and Protect Program. JMIR Pediatr Parent 4(2):e22822 33843603 10.2196/22822 PMC8076982

[31] Milton AC et al (2021) Technology-Enabled Reform in a Nontraditional Mental Health Service for Eating Disorders: Participatory Design Study. J Med Internet Res 23(2):e19532 33591283 10.2196/19532 PMC7925150

[32] McKercher K (2020) Beyond Sticky Notes. Co-Design for Real: Mindsets, Methods and Movements. Inscope Books, Sydney, Australia

[33] Thorburn K et al Creating the conditions for collaborative decision-making in co-design*.* CoDesign: pp. 1–18

[34] Löhr K, Weinhardt M, Sieber S (2020) The World Café as a Participatory Method for Collecting Qualitative Data. Int J Qualitative Methods 19:1609406920916976

[35] Harrison R et al (2024) Employing cofacilitation to balance power and priorities during health service codesign. Health Expect 27(1):e13875 37691618 10.1111/hex.13875 PMC10726141

[36] Lin C-P, Chiang C-H, Sheu S-J (2023) Using the Kipling method to explore the contextual factors of decision-making during advance care planning for older cancer patients, their family, and health-care professionals: A qualitative secondary analysis*.* Palliative and Supportive Care, : pp. 1–7 10.1017/S1478951523001256 37859416

[37] Someren M, Barnard Y, Sandberg J (1994) The Think Aloud Method - A Practical Guide to Modelling CognitiveProcesses

[38] Damschroder LJ et al (2009) Fostering implementation of health services research findings into practice: a consolidated framework for advancing implementation science. Implement Sci 4(1):50 19664226 10.1186/1748-5908-4-50 PMC2736161

[39] Pallesen KS et al (2020) A qualitative evaluation of participants’ experiences of using co-design to develop a collective leadership educational intervention for health-care teams. Health Expect 23(2):358–367 31999883 10.1111/hex.13002 PMC7104638

[40] Weiner BJ et al (2017) Psychometric assessment of three newly developed implementation outcome measures. Implement Sci 12(1):108 28851459 10.1186/s13012-017-0635-3 PMC5576104

[41] Australian Bureau of Statistics *Standards*. 03/09/2024]; Available from: https://www.abs.gov.au/statistics/standards

[42] Braun V, Clarke V (2019) Reflecting on reflexive thematic analysis. Qualitative Res Sport Exerc Health 11(4):589–597

[43] Byrne D (2022) A worked example of Braun and Clarke’s approach to reflexive thematic analysis, vol 56. Quality & Quantity, pp 1391–1412. 3

[44] Vaismoradi M, Turunen H, Bondas T (2013) Content analysis and thematic analysis: Implications for conducting a qualitative descriptive study. Nurs Health Sci 15(3):398–405 23480423 10.1111/nhs.12048

[45] Hsieh HF, Shannon SE (2005) Three approaches to qualitative content analysis. Qual Health Res 15(9):1277–1288 16204405 10.1177/1049732305276687

[46] Sekhon M, Cartwright M, Francis JJ (2017) Acceptability of healthcare interventions: an overview of reviews and development of a theoretical framework. BMC Health Serv Res 17(1):88 28126032 10.1186/s12913-017-2031-8 PMC5267473

[47] Bowen DJ et al (2009) How we design feasibility studies. Am J Prev Med 36(5):452–457 19362699 10.1016/j.amepre.2009.02.002 PMC2859314

[48] Michie S et al (2009) Specifying and reporting complex behaviour change interventions: the need for a scientific method. Implement Sci 4:40 19607700 10.1186/1748-5908-4-40 PMC2717906

[49] South Eastern Sydney Local Health District 27 September 2024]; Available from: https://www.seslhd.health.nsw.gov.au/keeping-body-mind

[50] Loopstra R (2018) Interventions to address household food insecurity in high-income countries. Proc Nutr Soc 77(3):270–281 29580316 10.1017/S002966511800006X

[51] Loopstra R, Tarasuk V (2012) The Relationship between Food Banks and Household Food Insecurity among Low-Income Toronto Families. Can Public Policy / Anal de Politiques 38(4):497–514

[52] Tarasuk V, Eakin JM (2005) Food assistance through surplus food: Insights from an ethnographic study of food bank work. Agric Hum Values 22(2):177–186

[53] McNaughton D et al (2021) Food Charity, Shame/ing and the Enactment of Worth. Med Anthropol 40(1):98–109 32717161 10.1080/01459740.2020.1776275

[54] Oldroyd L et al (2022) The nutritional quality of food parcels provided by food banks and the effectiveness of food banks at reducing food insecurity in developed countries: a mixed-method systematic review. J Hum Nutr Diet 35(6):1202–1229 35112742 10.1111/jhn.12994 PMC9790279

[55] Sherriff S et al (2022) Murradambirra Dhangaang (make food secure): Aboriginal community and stakeholder perspectives on food insecurity in urban and regional Australia. BMC Public Health 22(1):1066 35643511 10.1186/s12889-022-13202-z PMC9146813

[56] Chauhan A et al (2021) Optimising co-design with ethnic minority consumers. Int J Equity Health 20(1):240 34736455 10.1186/s12939-021-01579-z PMC8567634

[57] Weinstein LC et al (2021) It’s common sense that an individual must eat’: Advocating for food justice with people with psychiatric disabilities through photovoice. Health Expect 24(S1):161–173 32671916 10.1111/hex.13101 PMC8137492

[58] Åkerblom KB, Ness O (2023) Peer Workers in Co-production and Co-creation in Mental Health and Substance Use Services: A Scoping Review. Adm Policy Ment Health 50(2):296–316 36396756 10.1007/s10488-022-01242-x PMC9931804

[59] Sheikhan NY et al (2023) Exploring the impact of engagement in mental health and substance use research: A scoping review and thematic analysis. Health Expect 26(5):1806–1819 37282732 10.1111/hex.13779 PMC10485342

[60] Greenhalgh T, Papoutsi C (2019) Spreading and scaling up innovation and improvement. BMJ 365:pl2068 10.1136/bmj.l2068 PMC6519511 31076440

[61] Friesen P et al (2021) Measuring the impact of participatory research in psychiatry: How the search for epistemic justifications obscures ethical considerations. Health Expect 24(S1):54–61 31854081 10.1111/hex.12988 PMC8137501

[62] Harrison R et al (2023) Creating space for theory when codesigning healthcare interventions. J Eval Clin Pract 29(4):572–575 35700040 10.1111/jep.13720 PMC10947053

[63] Hecht AA et al (2018) Using a trauma-informed policy approach to create a resilient urban food system. Public Health Nutr 21(10):1961–1970 29458445 10.1017/S1368980018000198 PMC6088531

