# Supplementary material for: Food insecurity in adults living with severe mental illness in Australia: an exploration of causes and experiences using a co-design approach
Source: Soc Psychiatry Psychiatr Epidemiol. 2026 Apr 29;61(10):2081–95. doi: 10.1007/s00127-026-03082-8 (PMC13585879; doi:10.1007/s00127-026-03082-8)
Supplement: Supplementary file 1 [file 127_2026_3082_MOESM1_ESM.docx]

**Supplementary File 1**

Standards for Reporting Qualitative Research

| **No.** | | **Topic** | **Item** |
| --- | --- | --- | --- |
|  | | **Title and abstract** |  |
| S1 | | Title | P. 1 |
|  | |  |  |
|  | |  |  |
| S2 | | Abstract |  |
|  | |  | P. 2 |
|  | |  |  |
|  | | **Introduction** |  |
| S3 | | Problem formulation | P.4 |
|  | |  |  |
| S4 | | Purpose or research question | P.4 |
|  | | **Methods** |  |
|  | S5 | Qualitative approach and research paradigm | P. 4-7 |
|  |  |  |  |
|  |  |  |  |
|  |  |  |  |
|  | S6 | Researcher characteristics and reflexivity | P. 5-6 |
|  |  |  |  |
|  |  |  |  |
|  |  |  |  |
|  |  |  |  |
|  | S7 | Context | P.4 |
|  | S8 | Sampling strategy | P. 5 |
|  |  |  |  |
|  |  |  |  |
|  | S9 | Ethical issues pertaining to human subjects | P.4 |
|  |  |  |  |
|  |  |  |  |
|  | S10 | Data collection methods | P.4, 6 |
|  |  |  |  |
|  |  |  |  |
|  |  |  |  |
|  | S11 | Data collection instruments and technologies | P.5-6 |
|  |  |  |  |
|  |  |  |  |
|  | S12 | Units of study | P. 7 |
|  |  |  |  |
|  |  |  |  |
|  | S13 | Data processing | P. 6-7 |
|  |  |  |  |
|  |  |  |  |
|  |  |  |  |
|  | S14 | Data analysis | P. 6-7 |
|  |  |  |  |
|  |  |  |  |
|  | S15 | Techniques to enhance trustworthiness | P. 6-7 |
|  |  |  |  |
|  |  | **Results/findings** |  |
|  | S16 | Synthesis and interpretation | P.7-10 |
|  |  |  |  |
|  |  |  |  |
|  | S17 | Links to empirical data | P.7-10 |
|  |  |  |  |
|  |  | **Discussion** |  |
|  | S18 | Integration with prior work, implications, | P. 11 |
|  |  | transferability, and contribution(s) to the field |  |
|  |  |  |  |
|  |  |  |  |
|  |  |  |  |
|  | S19 | Limitations | P. 12 |

(*Table continues*)

| (Continued) |  | |
| --- | --- | --- |
| **No.** | **Topic** | **Item** |
|  | **Other** |  |
| S20 | Conflicts of interest | P. 3 |
| S21 | Funding | P. 3 |
