# Supplementary material for: Food insecurity in adults living with severe mental illness in Australia: an exploration of causes and experiences using a co-design approach
Source: Soc Psychiatry Psychiatr Epidemiol. 2026 Apr 29;61(10):2081–95. doi: 10.1007/s00127-026-03082-8 (PMC13585879; doi:10.1007/s00127-026-03082-8)
Supplement: Supplementary file 5 [file 127_2026_3082_MOESM5_ESM.docx]

**Supplementary File 3**

**Focus Group Guide**

- Introductions and acknowledgements
- Explain purpose of the focus group
- Introduce timing of questions (~65 minutes on model, ~25 minutes on co-design – 90 minutes total)
- Request permission to audio-record
- Answer any questions from participants; explain right to withdraw
- Disseminate and walk-through the intervention model

*Consolidated Framework for Implementation Research*

- How do you feel about the intervention being used in your setting (i.e., the Maroubra Centre)?
  - Why?

**15 MINUTE MARK**

- How does the intervention compare to other alternatives that may have been considered or that you know about?
  - For example, OzHarvest, soup kitchens, food pantries.
- How does the intervention compare to other similar existing programs in your setting?
  - For example, Keeping the Body in Mind?

**30 MINUTE MARK**

- What barriers will people face to participating in the intervention?
- How well does the intervention fit with existing practices in your setting?
- What kinds of changes or alterations do you think you will need to make to the intervention so it will work effectively in your setting?

**45 MINUTE MARK**

- What kinds of infrastructure changes will be needed to accommodate the intervention?
- How well do you think the intervention will meet the needs of the individuals served by your organisation?
- What kind of information should we also be collecting to determine if the intervention is effective?

**65 MINUTE MARK**

*Codesign*

- Could you describe what worked well during the codesign process?
- What aspects do you think didn’t work so well?
- Were there any challenges?
  - If so, what were they and what steps were taken to overcome these challenges?

**80 MINUTE MARK**

- Do you think everyone was engaged in the workshops, and did this change over time?
  - If not, what do you think might have prevented people from engaging with the workshops?
- Do you feel you benefited from participating in the workshops? If so, how?
- Is there anything else you would like to add about your experience that we haven’t asked about?

**90 MINUTE MARK**
